# Theoretical Insights into the Nature of Halogen Bonding in Prereactive Complexes

**DOI:** 10.1002/chem.201204312

**Published:** 2013-02-18

**Authors:** J Grant Hill, Xiaojun Hu

**Affiliations:** [a] School of Chemistry, Joseph Black Building, University of GlasgowGlasgow G12 8QQ (UK)

**Keywords:** ab initio calculations, halogen bonding, non-covalent interactions, theoretical chemistry

## Abstract

Benchmark quality geometries and interaction energies for the prereactive halogen-bonded complexes of dihalogens and ammonia, including hypothetical astatine containing dihalogens, have been produced via explicitly correlated coupled cluster methods. The application of local electron correlation partitioning reveals dispersion, electrostatics and ionic substitutions all contribute significantly to the interaction energy, with a linear relationship between the ionic substitutions and the degree of charge transfer. Potential energy curves for H_3_N⋅⋅⋅ClF show that as the relative orientations of the two subunits are manipulated appreciable interactions can be found at considerably angular displaced geometries, signifying lower directionality in halogen bonding than previously supposed.

## Introduction

Intermolecular halogen bonding is a form of non-covalent interaction between a halogen atom within a molecule and a second subunit, typically one with an electron-donating group. Although an IUPAC Task Group working on the definition of a halogen bond has yet to publish its final report, the similarity to the familiar hydrogen bond produces a working definition: “The halogen bond is an attractive interaction between a halogen atom X from a molecule or fragment R[001]X in which R is a group more electronegative than X or is X itself, and an atom or a group of atoms A in the same molecule R[001]X or in a different molecule B, where there is evidence of bond formation.”[1] In the intermolecular case, B is often a Lewis base and R can be another halogen atom or an (in)organic residue.

The earliest known example of a halogen-bonded complex dates to 1863,[2] but, apart from some notable exceptions,^3^–^5^ the 1990s saw the start of concerted efforts to characterise this type of interaction and exploit it in a number of scientific disciplines. Current practical applications of halogen bonding have been recently reviewed,[1] and these include the production of insulated supramolecular nanowires,[6] uses in rational drug design,[7] production of liquid crystals,[8] and crystal engineering.[9] Several theoretical investigations of halogen bonding systems have probed the electrostatic potentials of a number of R[001]X molecules and produced an insight into a property that is key for the ability to undergo halogen bonding.^10^–^13^ It has been shown that a region of positive potential is found as a “cap” on X, in a position such that it forms an extension of the R[001]X bond. Some groups refer to this electron-deficient region as a σ-hole,[14] and its presence allows for the intuitive description of halogen bonding as an electrostatic interaction between the Lewis base and the σ-hole.

A series of systematic experimental investigations into prereactive complexes of dihalogens (denoted XY herein, where X is the halogen atom directly involved in the intermolecular halogen bond) with Lewis bases by Legon and co-workers have produced detailed descriptions of halogen bonding in relatively small systems that are amenable to high-level ab initio theoretical studies.^1^,^15^,^16^ The main experimental technique used is known as pulsed-jet, Fourier-transform microwave spectroscopy, with the pulsed expansion and concentric capillaries ensuring the complexes are formed in a collisionless state. Freezing the interaction at the B⋅⋅⋅XY stage is crucial as the high reactivity of the lighter dihalogens can produce violent reactions, making observation of the halogen bond difficult. The present theoretical investigation is focused upon the halogen bonding between a dihalogen XY and ammonia as the Lewis base. The experimentally derived angular geometries of H_3_N⋅⋅⋅XY all show a *C*_3*v*_ structure,^17^–^22^ and this directionality conforms with what might be expected from the σ-hole concept; the optimal halogen bond should form such that R-X⋅⋅⋅N is linear to maximise the interaction between the area of electron depletion and the nitrogen lone pair.

In addition to geometrical information, rotational spectroscopy also provides data for the magnitude of the charge transfer between the two subunits undergoing halogen bonding. By recording the changes in the halogen nuclear quadrupole coupling constants that accompany halogen-bond formation it is possible to derive an expression for both the inter- (*δ*_i_) and intramolecular (*δ*_P_) electron transfer.[16] It has been demonstrated for a number of small Lewis bases that the magnitude of *δ*_i_ is small, with a maximum of approximately 0.14 of an electronic charge transferred from PH_3_ to ICl. Of relevance to the present study, the fractions of electronic charge transferred in H_3_N⋅⋅⋅Cl_2_, H_3_N⋅⋅⋅BrCl, and H_3_N⋅⋅⋅ICl are roughly 0.02, 0.06 and 0.08,^1^,^16^ respectively, indicating that a higher degree of polarisability in XY leads to greater electron transfer. Using the famous Mulliken notation,[23] this small amount of charge transfer would be termed an “outer complex” and it may be expected that the intermolecular interaction is weak. The experimental intermolecular stretching force constants (*k*_σ_) have also been determined for a number of H_3_N⋅⋅⋅XY complexes.^17^–^22^

Given the practical applications of halogen bonding, it is perhaps unsurprising that these non-covalent interactions have been the subject of a great number of theoretical studies (see refs. 11,24–34 for a few examples). In terms of the complexes considered in the current investigation, the highest level contributions are those of Karpfen,^35^–^37^ who used the second-order Møller-Plesset perturbation theory (MP2) and coupled cluster with single, double and perturbative triple excitations [CCSD(T)][38] methods to optimise geometries, dipole moments, polarisabilities, harmonic vibrational frequencies and interaction energies of the H_3_N⋅⋅⋅XY complexes, amongst others. A comparison of the MP2 and CCSD(T) interaction energies indicates that the MP2 method falls outside the “chemical accuracy” of 1 kcal mol^−1^ for most halogen bonding complexes and hence should be used only to uncover qualitative trends in halogen-bond interaction energies.

It is well-known that the accuracy of the coupled cluster methods depends strongly on the basis set used, and one of the goals of the present investigation is to use explicitly correlated (F12) coupled cluster methods to establish theoretical best-estimates for molecular geometries and interaction energies for the halogen bonding in H_3_N⋅⋅⋅XY complexes. These F12 methods significantly accelerate basis set convergence by including the interelectronic distance in the wave function,^39^,^40^ producing high-accuracy results with significantly reduced computational cost. Further insight into the nature of the halogen-bonding interaction in these systems is determined via an analysis of the fraction of electronic charge transferred between the subunits, and characterising how the interactions change when the halogen bond length and angle are manipulated. To the best of the authors’ knowledge, this includes high-level consideration of astatine in halogen bonding for the first time, and, although experimentally producing astatine complexes is somewhat unrealistic, it is hoped that this will provide useful additional insight into the nature of halogen bonding.

## Computational Methods

All calculations were carried out with the molpro^41^,^42^ package of ab initio programs. Coupled cluster calculations used the explicitly correlated CCSD(T)-F12b method,^43^,^44^ with the diagonal, fixed amplitude 3C(FIX) Ansatz.[45] Only the valence electrons were correlated. Two families of orbital basis sets were utilised within these calculations, the cc-pV*n*Z-F12[46] (referred to as V*n*Z-F12 herein) sets that were specifically designed for use in explicitly correlated calculations, and the aug-cc-pV*n*Z[47]–[49] (AV*n*Z herein) sets. For the post-d elements the recently developed cc-pV*n*Z-F12-PP orbital basis sets[50] matched to small-core relativistic pseudopotentials^51^,^52^ (PPs) were utilised. For the AV*n*Z calculations, the aug-cc-pV(*n*+d)Z basis sets that are specifically designed for second row elements were used for Cl.[53] Full technical details of the calculations are provided in the Supporting Information.

At the coupled cluster level basis set superposition error (BSSE) was compensated for by using the counterpoise (CP) method of Boys and Bernardi.[54] Geometry optimisations were also CP corrected and make no account for core-valence electron correlation or higher-order correlation effects. Estimates of CCSD(T)-F12b energies at the complete basis set (CBS) limit were produced using a Schwenke-type extrapolation[55]:

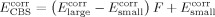
(1)
where 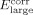 and 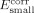 are the correlation energies evaluated with two systematically convergent basis sets, and *F* is, in this case, a previously optimised coefficient.[56] The CCSD-F12b and (T) contributions to the correlation energy are extrapolated separately, before summation with the HF reference energy (including CABS singles relaxation[57] as implemented in molpro)^58^,^59^ from the largest basis set calculation carried out. Two different energies of the interaction between the dihalogen and ammonia are presented. The first, termed interaction energy (IE), is the difference in energy between the prereactive complex and the energies of the two subunits fixed in their interacting geometries. The stabilisation energy is then defined as the difference in energy when the two subunits are in their isolated geometries (also referred to as the intrinsic bond energy).[60]

Local electron correlation (see ref. [61] for a recent review) calculations were carried out using the density fitted local MP2 correlation treatment (DF-LMP2, referred to as LMP2 herein)[62] with a density fitted Hartree–Fock (DF-HF) reference.^63^,^64^ Single-point LMP2 calculations were carried out using AVQZ basis sets, and further technical details are listed in the Supporting Information. CP corrections were not carried out at the LMP2 level as the local correlation treatment greatly reduces BSSE, and it seems reasonable to assume that any residual DF-HF BSSE will be negligible when calculated with large AVQZ basis sets.

## Results and Discussion

The halogen-bonding complexes considered in the present investigation are formed from dihalogens XY and ammonia (acting as a Lewis base). In order to establish the basis set dependence of these interactions and the geometries of the resulting supermolecules, a basis set convergence study is reported in the Supporting Information. CBS limit interaction energies and high-quality geometries are presented in the next section, before the amount of charge transferred between the subunits upon halogen bond formation is investigated. Finally, analysis of the interaction energy partitioning and how it varies with intermolecular halogen bond length and angle is in the final section.

### Geometries and interaction energies:

**Figure 1:**
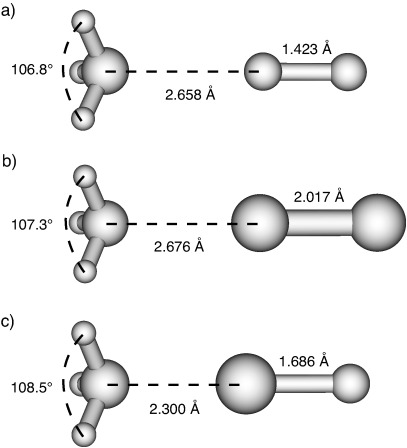
CP-CCSD(T)-F12b/VTZ-F12b optimised geometrical parameters of the prereactive halogen bonding complexes a) H_3_N⋅⋅⋅F_2_, b) H_3_N⋅⋅⋅Cl_2_, c) H_3_N⋅⋅⋅ClF. Full *Z*-matrices are provided in the Supporting Information.

The basis set convergence study presented in the Supporting Information, indicates that the AV*n*Z and V*n*Z-F12 families of basis sets produce almost identical results for both optimised geometries and interaction energies. V*n*Z-F12 is used herein due to better availability of auxiliary F12 basis sets for Br, I and At. The same study also shows that while the VDZ-F12 basis set performs well for intramolecular bond lengths and angles, the VTZ-F12 basis is required for a well-converged description of the intermolecular distance. Figure 1 illustrates the CP-CCSD(T)-F12b/VTZ-F12 optimised (*C*_3*v*_ symmetry) geometrical parameters for the H_3_N⋅⋅⋅F_2_, H_3_N⋅⋅⋅Cl_2_ and H_3_N⋅⋅⋅ClF complexes. The optimised N[001]H bond lengths are not shown, but they were found to be 1.012 Å in all cases.

Table 1 lists the optimised intermolecular and intrahalogen bond lengths for all combinations of halogens XY and XX. Initial geometry optimisations indicated that only complexes with the least electronegative halogen closest to ammonia are bound, that is, the least electronegative element is in the X position. This may be rationalised in terms of a σ-hole being produced on only the least electronegative atom for heteronuclear dihalogens, and is consistent with experimental findings. Comparison with the conventional CCSD(T)/aug-cc-pVTZ results of Karpfen indicates that the lower-level calculations produce intermolecular bond lengths that are too long by up to 0.06 Å,[37] and that MP2/aug-cc-pVTZ fortuitously produces geometries closer to CCSD(T)-F12b/VTZ-F12 than CCSD(T)/aug-cc-pVTZ does. The experimental estimates of the intermolecular bond lengths (2.708 Å for H_3_N⋅⋅⋅F_2_,[17] 2.73 Å for H_3_N⋅⋅⋅Cl_2_,[19] and 2.37 Å for H_3_N⋅⋅⋅ClF)[18] were obtained under the approximation that the monomer geometries do not change under complexation and are not directly comparable to the theoretical equilibrium geometry results in Figure 1, but it can be seen that the trends are much the same; the H_3_N⋅⋅⋅F_2_ intermolecular distance is only slightly shorter than that in H_3_N⋅⋅⋅Cl_2_, with H_3_N⋅⋅⋅ClF shorter by approximately 0.36 Å.

**Table 1:** Counterpoise corrected CCSD(T)-F12b/VTZ-F12 optimised intermolecular and intrahalogen bond lengths [Å] for the *C*_3*v*_ H_3_N⋅⋅⋅XY complexes. Full geometries are provided in the Supporting Information.

XY	*R*(N⋅⋅⋅X)	*R*(X[001]Y)
F_2_	2.658	1.423
Cl_2_	2.676	2.017
ClF	2.300	1.686
Br_2_	2.603	2.334
BrCl	2.529	2.194
BrF	2.333	1.821
I_2_	2.767	2.720
IBr	2.654	2.532
ICl	2.605	2.383
IF	2.496	1.959
At_2_	2.782	2.900
AtI	2.741	2.818
AtBr	2.666	2.625
AtCl	2.630	2.479
AtF	2.559	2.057

Estimates of the CCSD(T)-F12b/CBS interaction energies were produced at the VTZ-F12 CP-optimised geometries using the CCSD(T)-F12b/VTZ-F12 and CCSD(T)-F12b/VQZ-F12 single point energies, and are presented in Table 2. The interaction energies with each basis set are tabulated in the Supporting Information, and it can be seen that the interaction energy converges smoothly with basis set. Calculations at the VDZ-F12 level underestimate the CBS interaction energy by an average of 0.61 kcal mol^−1^, VTZ-F12 by 0.36 kcal mol^−1^, and VQZ-F12 by 0.13 kcal mol^−1^. All of these basis set incompleteness errors are within chemical accuracy of the CBS estimates. The CBS data also demonstrate that while the halogen bonding in H_3_N⋅⋅⋅F_2_ is quite weak, the interaction in the H_3_N⋅⋅⋅AtF complex is predicted to be approximately an order of magnitude greater at −20.32 kcal mol^−1^. In general, the interaction energies show that as the difference in electronegativity between the halogen elements X and Y increases, the strength of the interaction also increases. This is mirrored in Table 1 where an increase in electronegativity difference results in a shortening of both the intermolecular and intrahalogen bond lengths. A previous study on a subset of the dihalogens investigated here has noted a systematic increase in the strength of halogen bonding interaction with the polarisability of the halogen.[31]

**Table 2:** Counterpoise corrected CBS interaction energies [kcal mol^−1^] of H_3_N⋅⋅⋅XY halogen bonding complexes. All single point energy calculations were performed on CCSD(T)-F12b/VTZ-F12 optimised geometries. Experimental intermolecular force constants *k*_σ_ [N m^−1^] are listed for reference. See text for further details.

XY	CCSD(T)-F12b/CBS	Expt. *k*_σ_
F_2_	−1.83	4.7[17]
Cl_2_	−4.95	12.7[19]
ClF	−11.64	34.3[18]
Br_2_	−7.79	18.5[20]
BrCl	−9.67	26.7[21]
BrF	−16.65	–
I_2_	−8.20	–
IBr	−11.04	–
ICl	−12.91	30.4[22]
IF	−17.94	–
At_2_	−9.80	–
AtI	−11.24	–
AtBr	−14.10	–
AtCl	−15.99	–
AtF	−20.32	–

The results of Karpfen are referred to as both interaction energies and stabilisation energies in the original publication,[37] but a test calculation using Karpfen’s geometries suggests that the energies presented are analogous to those termed stabilisation energies in the current investigation (see the Supporting Information for more details). A comparison of the data from Karpfen’s investigation with the CBS stabilisation energies in the Supporting Information reveals that conventional CCSD(T)/aug-cc-pVTZ results can seriously underestimate the strength of the halogen bonding. For example, the H_3_N⋅⋅⋅BrF stabilisation energy is approximately 2.3 kcal mol^−1^ higher than the CBS estimate (−15.53 kcal mol^−1^) produced in the present investigation. This is entirely a basis set effect, as with a complete basis the CCSD(T) and CCSD(T)-F12b methods would produce identical results. While the interaction energies in Table 2 are not directly comparable to the experimental intermolecular force constants, it should be noted that they follow the same trend. For example, the force constant for H_3_N⋅⋅⋅Cl_2_ is roughly 2.7 times larger than that for H_3_N⋅⋅⋅F_2_, and the same is true for the calculated interaction energies. This suggests that if a H_3_N⋅⋅⋅AtF prereactive complex could be produced and analysed, the force constant should be around 50 N m^−1^.

Data in the Supporting Information show that as the halogen bond becomes stronger the relaxation energy (the difference between interaction and stabilisation energy) becomes larger. This is especially obvious for H_3_N⋅⋅⋅ClF where the relaxation energy is in excess of 1 kcal mol^−1^, and in all cases the relaxation energy has little basis set dependence. The corresponding effect of complexation on the intramolecular geometries is detailed in Table 3, where it can be seen that the formation of the intermolecular halogen bond increases the X[001]Y bond length and opens out the H-*N*-H angles in ammonia. The effect on the N[001]H bond lengths is negligible. It is noted that the experimental value of Δ*R*(X-Y) for H_3_N⋅⋅⋅Cl_2_ is +0.014 Å,[19] which is slightly shorter than the theoretical value in Table 3.

**Table 3:** Change in CP-CCSD(T)-F12b/VTZ-F12 geometries on formation of H_3_N⋅⋅⋅XY. Bond lengths in Å and angles in degrees.

XY	Δ*R*(X-Y)	Δ*R*(N-H)	Δ*θ*(HNH)
F_2_	+0.012	0.000	+0.18
Cl_2_	+0.025	0.000	+0.67
ClF	[0.057	−0.001	[1.91
Br_2_	[0.042	0.000	[1.24
BrCl	[0.051	0.000	[1.39
BrF	[0.060	0.000	[1.90
I_2_	[0.041	0.000	[1.03
IBr	[0.052	0.000	[1.29
ICl	[0.057	0.000	[1.34
IF	[0.049	0.000	[1.49
At_2_	[0.042	0.000	[1.00
AtI	[0.048	0.000	[1.03
AtBr	[0.055	0.000	[1.15
AtCl	[0.059	0.000	[1.16
AtF	[0.047	0.000	[1.20

### Degree of charge transfer:

The amount of intermolecular electron transfer on formation of H_3_N⋅⋅⋅XY has been assessed via the natural bond orbital (NBO) method.[65] The NBOs were obtained using the CCSD density matrix calculated with the aug-cc-pV(T+d)Z basis set,[53] and the degree of electron transfer was subsequently determined from NBO population analysis of the interacting complex and two subunits. For bromine and heavier halogens the aug-cc-pVTZ-PP basis sets and pseudopotentials were used.^52^,^66^,^67^ NBO population analyses on some of these systems have been carried out in the past, and it has been noted that there is an almost linear relationship between the magnitude of the interaction energy and the fraction of an electronic charge transferred on halogen bond formation (negative charge is transferred from ammonia to the dihalogen).^26^,^31^ The current NBO calculations use a higher level method and basis set throughout, and for the first time include astatine containing dihalogens.

**Figure 2:**
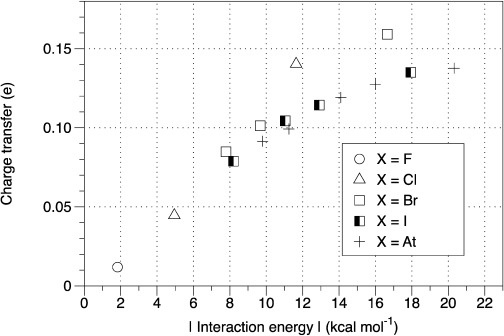
The fraction of an NBO derived electronic charge transferred from H_3_N to XY on formation of H_3_N⋅⋅⋅XY plotted against the interaction energy.

Figure 2 plots the fraction of an electronic charge transferred against the CBS interaction energy for different permutations of H_3_N⋅⋅⋅XY, with the symbols indicating the identity of halogen X. The fractions of an electronic charge transferred are also tabulated in the Supporting Information. It can be seen that the correlation between charge transfer and interaction energy is almost linear for the lighter halogens, but this begins to tail off when X=I or At. This change in trend is further investigated in terms of correlation energy partitioning in the next section. The position of ClF also appears to be something of an outlier in the lighter halogens, with a greater amount of charge transfer than might be expected from the interaction energy. The NBO derived charge transfer can be compared to the experimental *δ*_i_ values, and it is observed that theoretical values are a little larger than experimental, for example, on formation of H_3_N⋅⋅⋅BrCl *δ*_i_ is roughly 0.06 of an electronic charge, with a corresponding theoretical value of 0.10. Nevertheless, there is good qualitative agreement in all cases where experimental data is available, and Mulliken outer-type complexes are indicated throughout.

**Figure 3:**
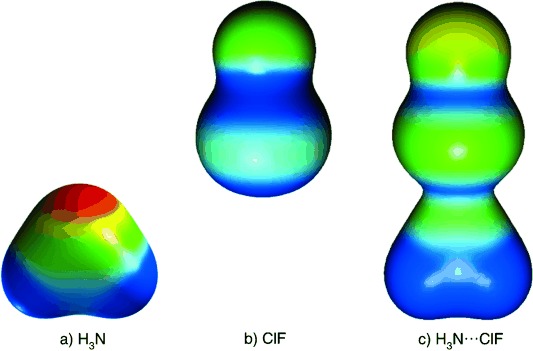
Isodensity surfaces (0.02 a.u.) colour-coded with the MP2/aug-cc-pVDZ electrostatic potential. Red indicates an electrostatic potential less than −0.01 a.u., blue greater than +0.01 a.u., and green between −0.05 and +0.05 a.u. Plots created using molden.[69]

Further qualitative evidence of both the charge transfer and electrostatic interactions between ammonia and XY can be seen in Figure 3, where isodensity surfaces colour-coded with the electrostatic potential are plotted for H_3_N, ClF, and H_3_N⋅⋅⋅ClF. The negatively charged cap on isolated ammonia corresponds to the lone pair, and the area of positive charge on chlorine is the σ-hole. It can be seen that on formation of H_3_N⋅⋅⋅ClF an area of somewhat negative charge (yellow/orange) is located on fluorine, with the negative charge associated with the lone pair no longer visible.

### Interaction energy partitioning:

Insights into the nature of the interactions between the dihalogen XY and the Lewis base NH_3_ can be obtained from a partitioning of the LMP2 interaction energy into contributions from different classes of excitations. Specifically, these are intramolecular correlation (*E*_intra-corr_) effects, dispersive coupling (*E*_disp_), dispersion exchange (*E*_disp-exch_), and ionic (*E*_ionic_) substitutions.[68] These contributions can be summed with the HF interaction energy (Δ*E*_SCF_) to give a total LMP2 interaction energy. Table 4 displays the partitioning according to these different excitation classes, with the LMP2 single point calculations carried out on top of the CP-CCSD(T)-F12b/VTZ-F12 optimised geometries. The partitioning could not be carried out for XY=At_2_ and AtI as in these two cases the intramolecular bond between the halogen atoms is longer than the intermolecular halogen bond (see Table 1), preventing the correct detection of the subunits by the partitioning algorithm.

**Table 4:** Partitioning of LMP2/AVQZ interaction energy [kcal mol^−1^] of H_3_N⋅⋅⋅XY halogen bonded complexes, see text for further details. Interaction energy is equal to the sum of the other contributions.

XY	Δ*E*_SCF_	*E*_intra-corr_	*E*_disp_	*E*_disp-exch_	*E*_ionic_	IE
F_2_	+0.30	+0.25	−1.17	+0.00	−1.32	−1.93
Cl_2_	−1.01	+1.79	−2.60	+0.05	−3.46	−5.23
ClF	−5.50	+6.64	−3.44	+0.25	−10.37	−12.41
Br_2_	−2.30	+2.67	−3.17	+0.15	−5.64	−8.28
BrCl	−3.96	+3.65	−3.35	+0.20	−6.83	−10.29
BrF	−10.82	+7.61	−3.92	+0.19	−10.44	−17.34
I_2_	−3.32	+2.71	−3.17	+0.12	−4.94	−8.60
IBr	−5.91	+4.09	−3.46	+0.13	−6.38	−11.54
ICl	−7.77	+4.72	−3.58	+0.12	−6.95	−13.46
IF	−13.21	+6.86	−4.05	−0.05	−7.86	−18.31
At_2_	−4.89	–	–	–	–	−10.22
AtI	−6.47	–	–	–	–	−11.70
AtBr	−9.33	+4.48	−3.62	+0.08	−6.22	−14.61
AtCl	−11.23	+4.87	−3.71	+0.05	−6.47	−16.50
AtF	−16.11	+6.65	−4.01	−0.09	−6.91	−20.48

A comparison of the LMP2 interaction energies with the CBS results reported in Table 2 reveals that LMP2 reproduces the more accurate interaction energies quite well, being overbound by an average of 0.46 kcal mol^−1^. This contrasts strongly with the results of Karpfen, where MP2 stabilisation energies were typically greater than 1 kcal mol^−1^ more strongly bound than CCSD(T).[37] There are two main factors for this; firstly, the present LMP2 calculations were carried out at a CCSD(T)-F12b geometry and not at the LMP2 minimum. Secondly, both the MP2 and CCSD(T) energies of Karpfen are further from convergence with respect to basis set than the results from the current investigation.

Analysing the components of the interaction energy in Table 4 shows that, for all halogens apart from F_2_, the HF interaction energy is attractive and forms a relatively large contribution to the overall interaction energy. As this Δ*E*_SCF_ term includes the electrostatic energy that is postulated to play a large role in the halogen bonding of such complexes,[15] this is largely unsurprising, yet provides some insight into why the halogen bond in H_3_N⋅⋅⋅F_2_ is weak. The correlation correction to the electrostatic energy forms part of *E*_intra-corr_ in LMP2 partitioning (along with correlation corrections to induction energy and exchange repulsion), which is repulsive for all of the H_3_N⋅⋅⋅XY systems. The *E*_disp-exch_ term is small in all cases, but the dispersive coupling contribution is attractive, and for all but the lightest dihalogens it is relatively constant at 3–4 kcal mol^−1^.

**Figure 4:**
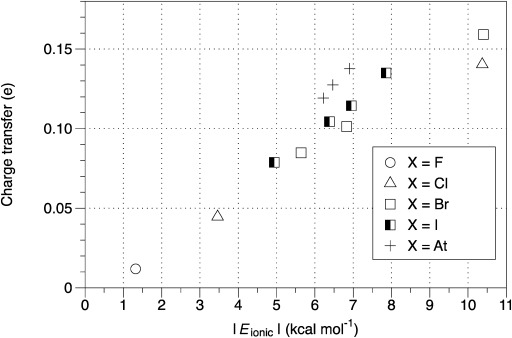
The fraction of an electronic charge transferred from H_3_N to XY on formation of H_3_N⋅⋅⋅XY plotted against the magnitude of the ionic substitution contribution to the LMP2 interaction energy (*E*_ionic_).

It can also be seen that the second most important term in the overall interaction energy is the ionic substitutions, implying that this term is significant in the formation of halogen bonds. This class of excitation represents the promotion of one electron from an occupied orbital on one subunit to the virtual orbital space associated with the second subunit, and is accompanied by a single excitation located solely on the second subunit. Hence, this type of double excitation sees electronic charge transferred from one subunit to the other and a plot of the NBO derived electronic charge transfer against *E*_ionic_ is shown in Figure 4. The relationship between the charge transfer and *E*_ionic_ is demonstrated to be almost linear, with a coefficient of determination (*R*^2^) of 0.86. If the three astatine containing dihalogens, which visually appear to be outliers, are removed from the set the correlation becomes significantly stronger with *R*^2^ = 0.95. This again suggests that the halogen bonding interaction is subtly different for these heavy halogen containing complexes.

The discussion of Figure 2 above noted that the formation of H_3_N⋅⋅⋅ClF resulted in a larger amount of charge transfer than might be expected from the magnitude of the interaction energy. Further evidence for this can be seen in Table 4, where it is shown that the H_3_N⋅⋅⋅ClF interaction energy has a large *E*_ionic_ component, representing 84% of the total interaction energy compared to somewhere between 50–70% for most of the other complexes. It could be reasoned that this is due to ClF being the heteronuclear dihalogen with the two most electronegative elements. The high electronegativity of fluorine withdraws electron density from chlorine, which in turn is also highly electronegative resulting in more charge transfer from ammonia. It is noted that the repulsive *E*_intra-corr_ term is also relatively large for this system. Figure 2 also shows that the relationship between charge transfer and interaction energy deviates away from linearity for some of the heavier halogens, particularly for XY=IF, AtBr, AtCl, and AtF. These same systems are also furthest from the linear trend of charge transfer against *E*_ionic_ in Figure 3, and it can be seen from Table 4 that the LMP2 interaction energies have relatively small *E*_ionic_ contributions (approximately 40% or smaller) along with large Δ*E*_SCF_ contributions, providing some insight into the subtly different interactions for these heavier dihalogens. This is also consistent with a previous investigation of formaldehyde⋅⋅⋅halomethane complexes, where it was demonstrated that the interaction in iodine containing complexes was dominated by electrostatic interactions, but the interaction in lighter halogens was principally dispersive.[11]

**Figure 5:**
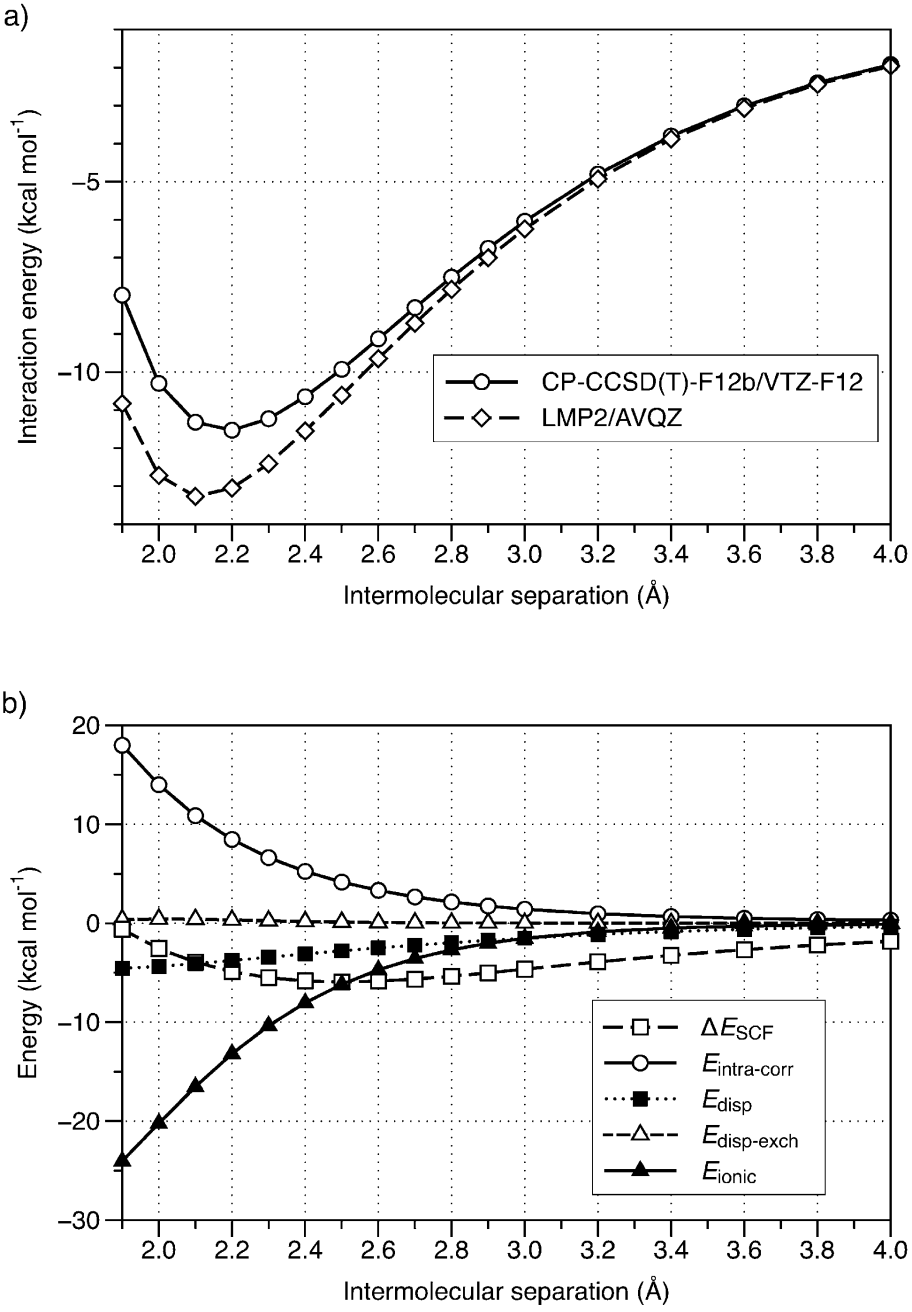
Potential energy scan of the intermolecular separation in the H_3_N⋅⋅⋅ClF halogen bonded complex. a) Comparison of the CP-CCSD(T)-F12b/VTZ-F12 and LMP2/AVQZ interaction energies. b) Partitioning of the LMP2/AVQZ interaction energy. CP-CCSD(T)-F12b/VTZ-F12 geometries are used throughout, see text for further details.

In order to examine the dependence of halogen bonding on distance and angle, energy partitioning has been carried out on one-dimensional cuts of the H_3_N⋅⋅⋅ClF potential energy surface that vary the intermolecular bond length and a H-*N*-Cl angle. The individual geometries were optimised at the CP-CCSD(T)-F12b/VTZ-F12 level by fixing either the bond length or angle and relaxing all other internal coordinates. Figure 5 displays the interaction energies for the relaxed potential energy scan of the intermolecular separation, along with the partitioning of the energy at the same points. It can be seen that, in the region of the minima, LMP2 overestimates the strength of the interaction by up to 1.74 kcal mol^−1^, but it also predicts a minimum with a slightly shorter intermolecular halogen bond length. Despite these differences, examining the partitioning of the interaction energy will provide some qualitative insight into the nature of the halogen bonding interaction as the intermolecular bond is artificially stretched. Figure 5b shows that as the bond is compressed the ionic contribution becomes strongly attractive, while simultaneously the intramolecular correlation effects become strongly repulsive. The *E*_disp-exch_ term is essentially negligible for all halogen bond lengths considered, while *E*_disp_ is attractive. As may be expected, all of the correlation energy components rapidly approach zero as the halogen bond is stretched, with the Δ*E*_SCF_ term (i.e., electrostatics) accounting for the attractive total interaction energy at long distance (beyond around 3.2 Å).

**Figure 6:**
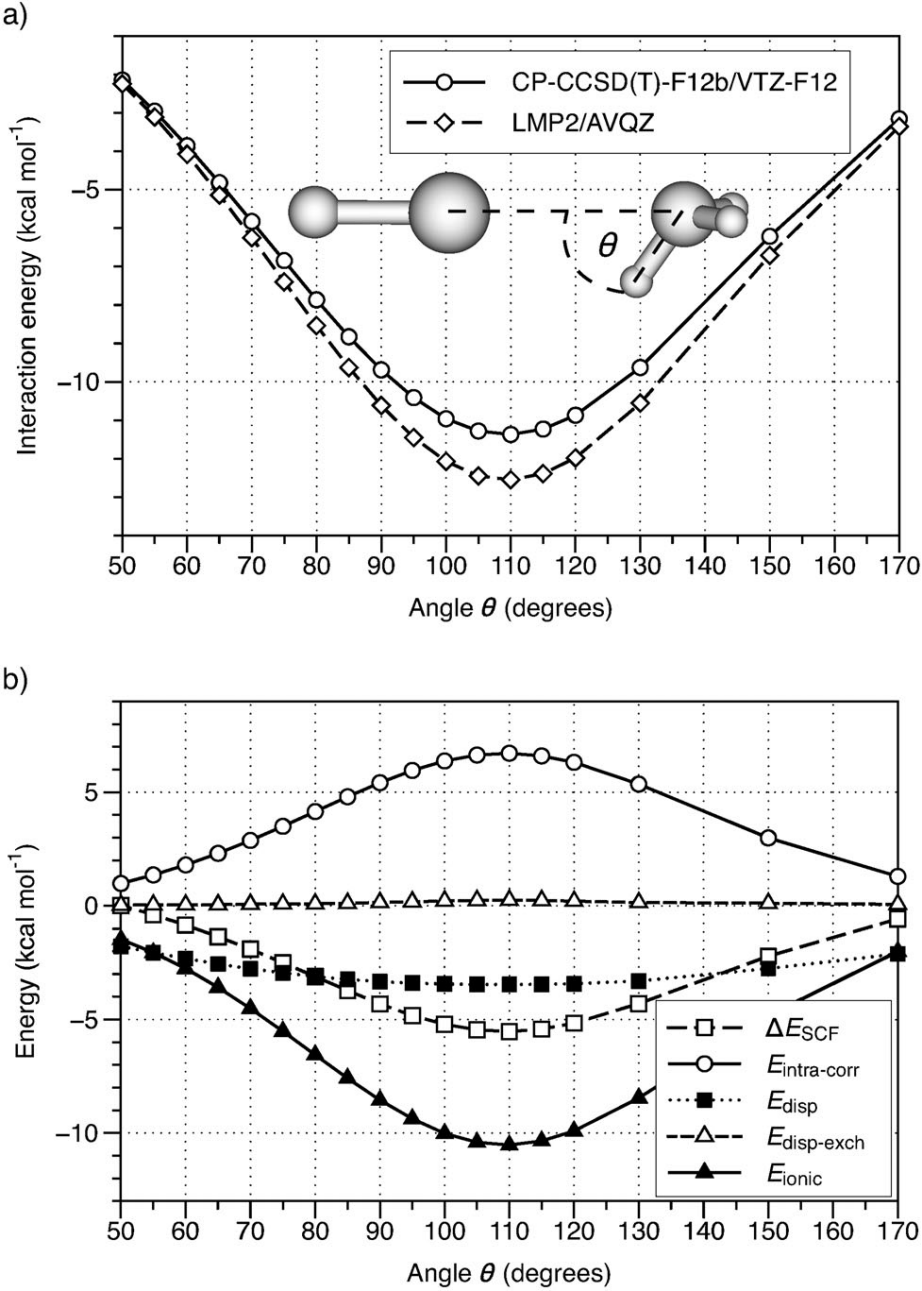
Potential energy scan of the H-*N*-Cl angle (*θ*) in the H_3_N⋅⋅⋅ClF halogen bonded complex. a) comparison of the CP-CCSD(T)-F12b/VTZ-F12 and LMP2/AVQZ interaction energies. b) Partitioning of the LMP2/AVQZ interaction energy. CP-CCSD(T)-F12b/VTZ-F12 geometries are used throughout, see text for further details.

The interaction energies resulting from the relaxed potential energy scan of the H-*N*-Cl angle (*θ*) are presented in Figure 6a, which also includes a diagram visualising *θ*. Imagining a simple Lewis-like structure, varying this angle moves the lone pair of electrons away from a linear extension of the intramolecular bond between the halogen atoms and thus acts as a probe of the directionality of the intermolecular halogen bond. As expected, for both methods the minimum in the interaction energy occurs at the tetrahedral angle, corresponding to *C*_3*v*_ symmetry and the ammonia lone pair forming a linear extension to XY. LMP2 overestimates the strength of the halogen bond by 1.18 kcal mol^−1^, but it can be seen that the two interaction energies become much closer as the angle moves away from tetrahedral. It is perhaps surprising that the interaction energy curve has a relatively shallow gradient, for example, when *θ*=90° the IE is roughly −10 kcal mol^−1^, and at *θ*=65° it is approximately −5 kcal mol^−1^. This suggests that although the halogen bond prefers to adopt the *C*_3*v*_ geometry, there is still appreciable attractive interactions occurring at relatively perturbed angles and it may be possible to exploit this reduced directionality in the practical applications of halogen bonding.

Some insights into the forces involved in the halogen bond at different values of *θ* can be obtained from Figure 6b, which shows the LMP2 partitioning of the interaction energy. As to be expected, the *E_disp_* contribution has no dependence on *θ* and the slight variations in this term may be attributed to changes in the intermolecular distance as the geometry is relaxed at a fixed angle. The Δ*E*_SCF_ and *E*_ionic_ contributions both pass through minima at the geometry corresponding to the interaction energy minimum, with a maximum in the repulsive *E*_intra-corr_ term. As *θ* approaches 50° most of the contributions are close to zero, with only *E*_disp_ and *E*_ionic_ accounting for most of the interaction energy. It appears that the sum of *E*_ionic_ and *E*_disp_ dominates the interaction throughout, with the sum of Δ*E*_SCF_ and *E*_intra-corr_ relatively constant at approximately +1 kcal mol^−1^. Figure S2 in the Supporting Information shows that there is again a linear relationship between the NBO derived charge transfer and the magnitude of *E*_ionic_ for different values of *θ*. A possible interpretation of the interaction energy partitioning could be that when the angle is varied the electrostatic contribution is small and the halogen bond is composed mostly of dispersion and charge transfer, which is somewhat different to the bond stretching case. It can be expected that other dihalogens will display similar trends, recalling the differences in Table 4, and this is evidenced by the H_3_N⋅⋅⋅BrCl potential energy scans and partitioning presented in Figures S4 and S5 in the Supporting Information.

## Conclusion

Given the numerous computational studies of halogen bonding, there has been surprisingly little effort made to establish benchmark quality interaction energies on prototypical systems such as dihalogens bound to ammonia. The advent of the explicitly correlated F12 methods and associated basis sets allows for the efficient production of such reference data and CP-CCSD(T)-F12b/CBS interaction and stabilisation energies have been presented for all possible dihalogens, including hypothetical astatine permutations. Previous, lower-level, studies have demonstrated that an increase in polarisability of the dihalogen produces an increase in interaction energy. The current work supports these findings and demonstrates that this trend also applies to the astatine containing molecules. A comparison with previous, conventional coupled cluster calculations indicates that F12 methods are necessary in the production of reference-quality data as convergence of the interaction energy with respect to basis set is slow.

A combination of NBO analysis and local electron correlation partitioning has provided some additional insight into the nature of the non-covalent interaction. Previous experimental and theoretical investigations had noted that the overall charge transferred from ammonia to the dihalogen upon complexation is a relatively small fraction of an electron (on the order of 0.1), but there is a correlation between the amount of charge transfer and the strength of the interaction. The current investigation adds further weight to this correlation and reveals that the ClF dihalogen appears to have a larger than expected degree of charge transfer, while the heavier halogen containing complexes tend to have higher interaction energies than one might expect from the charge transferred. A linear correlation was also found between the ionic substitution excitation contribution and the degree of charge transfer, which appears significant as this is a major contribution to the overall interaction energy. As may be expected from the σ-hole concept, the HF contribution to the interaction energy, and therefore electrostatic interactions, also provides a large contribution to the more polarisable dihalogens.

It is relatively well-known that H_3_N⋅⋅⋅F_2_ is a weak interaction, with the present analysis revealing that this is due to a repulsive HF component and a very small amount of charge transfer. Dispersion interactions provide more than 50 % of the attractive interaction energy. It has also been demonstrated that the astatine-containing (and some iodine-containing) complexes have a subtly different halogen bonding interaction; when compared to other complexes there is a smaller degree of charge transfer/ionic substitution and a larger HF contribution. With the exception of the F_2_ complex, the magnitude of the dispersion contribution remains relatively constant for all systems under investigation.

Adjusting the angle formed between H-*N*-X, and hence the relative orientation of the ammonia lone pair and the σ-hole, acts as a probe of the directionality of the halogen bond. For the ClF dihalogen it has been shown that there is still an appreciable attractive interaction of 5 kcal mol^−1^ at an angle of 65°, a considerable deviation from the *C*_3*v*_ minimum. Partitioning of the interaction energy reveals that dispersion forces contribute almost as much as charge transfer at large angle displacements, with little contribution from electrostatics. This is in complete contrast to partitioning of the interaction as the intermolecular halogen bond is stretched. Strong directionality is a commonly-held belief for halogen-bonding interactions, meaning that the current results of a relatively shallow gradient potential energy curve may have important implications in practical applications such as crystal engineering and rational drug design. Further investigations are currently underway to discover how these findings translate to larger halogen bonded systems.

## Notes

As a service to our authors and readers, this journal provides supporting information supplied by the authors. Such materials are peer reviewed and may be re-organized for online delivery, but are not copy-edited or typeset. Technical support issues arising from supporting information (other than missing files) should be addressed to the authors.
