# Supplementary material for: Theoretical Insights into the Nature of Halogen Bonding in Prereactive Complexes
Source: Chemistry. 2013 Feb 18;19(11):3620–8. doi: 10.1002/chem.201204312 (PMC3790957; doi:10.1002/chem.201204312)
Supplement: Supplementary file 1 [file chem0019-3620-SD1.pdf]

CHEMISTRY
A EUROPEAN JOURNAL

Supporting Information

© Copyright Wiley-VCH Verlag GmbH & Co. KGaA, 69451 Weinheim, 2013

Theoretical Insights into the Nature of Halogen Bonding in Prereactive Complexes

J. Grant Hill* and Xiaojun Hu[a]

chem_201204312_sm_miscellaneous_information.pdf

1 Computational procedure

All calculations were carried out with the MOLPRO[1, 2] package of *ab initio* programs. Coupled cluster calculations used the explicitly correlated CCSD(T)-F12b method,[3, 4] with the diagonal, fixed amplitude 3C(FIX) *Ansatz*.[5] Only the valence electrons were correlated. Two families of orbital basis sets were utilised within these calculations, the cc-pVnZ-F12[6] (referred to as VnZ-F12 herein) sets that were specifically designed for use in explicitly correlated calculations, and the aug-cc-pVnZ[7] (AVnZ herein) sets. For systems involving chlorine atoms the aug-cc-pV(n+d)Z sets containing an additional tight-d function were employed in AVnZ. These basis sets are only available for second-row elements and have been demonstrated to produce significantly better results than aug-cc-pVnZ.[8] For the post-d elements the recently developed cc-pVnZ-F12-PP orbital basis sets[9] and small-core relativistic pseudopotentials[10, 11] (PPs) were utilised. Density fitting of the Fock and exchange matrices used the def2-QZVPP/JKFit[12] auxiliary basis sets (ABSs) for the post-d elements, and cc-pVnZ/JKFit[13] elsewhere. Density fitting of the remaining two electron integrals was performed with the VnZ-F12-PP/MP2Fit[9] sets for post-d elements and AVnZ/MP2Fit[14, 15] for all other elements. The OptRI compact ABSs[9, 16, 17] matched to specific orbital basis sets were used in the CABS procedure[18] for the RI approximation. Recommendations for the combination of orbital and auxiliary basis sets are detailed in previous work.[19] The geminal Slater exponent (β) was set to values recommended elsewhere,[4, 19] namely 0.9, 1.0 and 1.0 a_0^{-1} for VDZ-F12, VTZ-F12 and VQZ-F12, respectively, and 1.0, 1.2 and 1.4 for AVDZ, AVTZ and AVQZ, respectively.

At the coupled cluster level basis set superposition error (BSSE) was compensated for by using the counterpoise (CP) method of Boys and Bernardi.[20] Geometry optimisations were also CP corrected by minimising the quantity

$$E_{\text{opt}} = E_{\text{dimer}} - E^{\text{inter}}_{\text{mon1}} - E^{\text{inter}}_{\text{mon2}} + E^{\text{sep}}_{\text{mon1}} + E^{\text{sep}}_{\text{mon2}}, \tag{1}$$

where all monomer calculations are carried out in the basis set of the full system. $E^{\text{inter}}_{\text{mon}x}$ denotes that the calculation was performed with ghost atoms in the interacting system geometry and $E^{\text{sep}}_{\text{mon}x}$ indicates that the energy was evaluated with the ghost atoms corresponding to the other monomer placed at a distance of 1000 Å (such that they do not interact).[21] The relaxation energy of the complexes was obtained by optimising the geometries of the isolated monomers and computing the quantity

$$E_{\text{relax}} = E^{\text{free}}_{\text{mon1}} + E^{\text{free}}_{\text{mon2}} - E^{\text{sep}}_{\text{mon1}} - E^{\text{sep}}_{\text{mon2}}, \tag{2}$$

which can then be subtracted from the CP corrected interaction energy (IE) to give a CP corrected interaction energy relative to relaxed monomers. This quantity is known as the stabilisation energy (SE), which can be related to the experimentally observable dissociation energy *via* the addition of vibrational zero-point energy differences.[22] The convergence criteria for the geometry optimisation using numerical gradients utilised were the MOLPRO defaults, with the exception of the required accuracy of the optimised gradient, which was set to 1×10^{-4}.

Local electron correlation (see Ref. [23] for a recent review) calculations were carried out using the density fitted local MP2 correlation treatment (DF-LMP2, referred to as LMP2 herein)[24] with a density fitted Hartree-Fock (DF-HF) reference.[13, 25] Single point LMP2 calculations were carried out using AVQZ basis sets, including aug-cc-pVQZ-PP[11, 26, 27] with relativistic small-core PPs[10, 11, 27] for the post-d elements. The DF-HF reference employed the cc-pVQZ/JKFit and def2-QZVPP/JKFit ABSs,[12, 13] and the aug-cc-pVQZ/MP2Fit and aug-cc-pVQZ-PP/MP2Fit[28] ABSs were used in the correlation treatment. The most diffuse function in each angular momentum symmetry were eliminated in the Pipek-Mezey orbital localisation[29] and the orbital domain selection used the Boughton-Pulay method[30] with a completeness criterion of 0.990. The minimum Mulliken charge for a hydrogen atom to be included in a domain was set to 0.15. Domains were determined at large intermolecular separation and frozen for the interacting system. Partitioning of the interaction energy into

different excitation classes was carried out,[31] providing intramolecular-correlation, dispersion, dispersion-exchange and ionic terms. CP corrections were not carried out at the LMP2 level as the local correlation treatment is BSSE-free by construction, and it seems reasonable to assume that any residual DF-HF BSSE will be negligible when calculated with large AVQZ basis sets.

2 Basis set convergence

While there have been a number of previous theoretical investigations into halogen bonding in $H_3N\cdots XY$ complexes, existing coupled cluster level studies have been limited to the aug-cc-pVTZ basis set.[32] Hence, little is known about the high-level basis set dependence of the interaction energies and geometries of these complexes. Fig. S1 illustrates the optimised (constrained to C_{3v} point group) geometrical parameters for $H_3N\cdots F_2$, $H_3N\cdots Cl_2$ and $H_3N\cdots ClF$ complexes, with the interaction energies of the same complexes presented in Table S1. Optimised N–H bond lengths are not shown as they are largely basis set independent (generally 1.012 Å). These complexes were selected to test the basis set convergence as OptRI ABSs are available that are matched to both the VnZ-F12 and AVnZ families of basis sets, which is not the case for the heavier halogens.

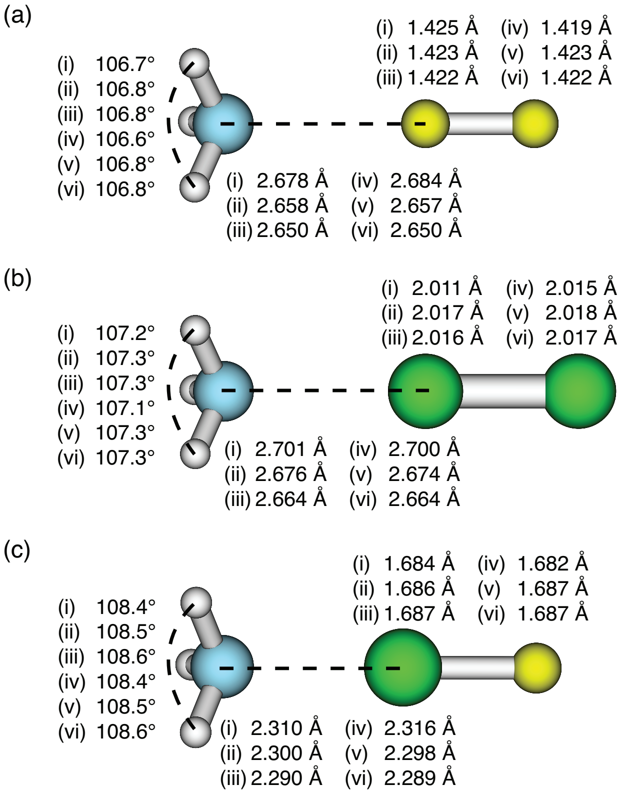

Figure S1: CP-CCSD(T)-F12b optimised geometrical parameters of the prereactive halogen bonding complexes (a) $H_3N\cdots F_2$, (b) $H_3N\cdots Cl_2$, (c) $H_3N\cdots ClF$. The basis sets used are (i) = VDZ-F12, (ii) = VTZ-F12, (iii) = VQZ-F12, (iv) = AVDZ, (v) = AVTZ, (vi) = AVQZ.

Fig. S1 shows that, as may be expected, the convergence of the CCSD(T)-F12b intramolecular bond lengths and angles is very rapid, with DZ results within 0.005 Å and 0.2° of QZ. Increasing the basis set size to TZ quality places the bond lengths within 0.001 Å of QZ. However, Fig. S1 also shows that the basis set effect is around an order of magnitude larger for the intermolecular bond length, with DZ results between 0.02 and 0.04 Å longer than QZ, TZ reduces this to around 0.01 Å. In all cases, the intermolecular halogen bond distance decreases as the basis

Table S1: Counterpoise corrected CCSD(T)-F12b interaction energies (kcal mol^{-1}) of lighter $H_3N\cdots XY$ complexes. Stabilisation energy (SE) gives the CP corrected interaction energy relative to the relaxed monomers. See text for further details

XY		VDZ-F12	VTZ-F12	VQZ-F12	AVDZ	AVTZ	AVQZ
F_2	CP-IE	-1.69	-1.74	-1.79	-1.68	-1.77	-1.80
	BSSE	0.13	0.06	0.03	0.31	0.10	0.05
	SE	-1.64	-1.69	-1.73	-1.63	-1.72	-1.75
Cl_2	CP-IE	-4.47	-4.75	-4.87	-4.59	-4.80	-4.92
	BSSE	0.28	0.11	0.06	0.36	0.18	0.09
	SE	-4.33	-4.59	-4.70	-4.44	-4.64	-4.74
ClF	CP-IE	-10.87	-11.24	-11.53	-10.78	-11.30	-11.57
	BSSE	0.37	0.15	0.07	0.57	0.25	0.13
	SE	-9.83	-10.16	-10.40	-9.75	-10.21	-10.43

set size is increased, and there is excellent agreement between equilibrium geometries optimised using the VnZ-F12 and AVnZ families of basis sets.

The CP corrected interaction energies in Table S1 show that as the quality of the basis set is increased, the strength of the interaction increases. The magnitude of this increase depends greatly on the system in question, with a small, 0.1 kcal mol^{-1}, increase in strength noted for $H_3N\cdots F_2$, but a significantly larger (0.66 kcal mol^{-1}) increase observed on going from VDZ-F12 to VQZ-F12 for $H_3N\cdots ClF$. The increase seen from TZ to QZ is still somewhat significant for the latter system, at around 0.3 kcal mol^{-1}. The AVnZ family of basis sets produces slightly lower, and presumably more accurate, interaction energies than VnZ-F12 at the TZ and QZ levels, perhaps indicating the effect of higher angular momentum diffuse functions in AVnZ (it should be noted that the VnZ-F12 basis sets include diffuse s and p functions). The difference between these values is less than 0.1 kcal mol^{-1}, and thus there is little to choose between them for practical purposes. At the DZ level there is a slight preference for VnZ-F12, which may be caused by its larger s and p core. It should also be noted that whilst BSSE is low for all basis sets and systems investigated, the VnZ-F12 family may be preferred if CP corrections are not carried out.

Table S1 shows that as the halogen bond becomes stronger the relaxation energy (the difference between CP-IE and SE) becomes larger, which is especially obvious for $H_3N\cdots ClF$ where the relaxation energy is in excess of 1 kcal mol^{-1}. This relaxation energy has little basis set dependence. The effect of complexation on the intramolecular geometries is detailed at the CCSD(T)-F12b/VQZ-F12 level in Table S2, where it can be seen that the formation of the intermolecular halogen bond increases the X–Y bond length and opens out the H–N–H angles in ammonia. The effect on the N–H bond lengths is negligible. Logically, stronger intermolecular interactions produce larger changes in the intramolecular geometries of the subunits. It is noted that the experimental value of ΔR(X–Y) for $H_3N\cdots Cl_2$ is +0.014 Å,[33] which is slightly shorter than the theoretical value in Table S2.

Table S2: Change in CCSD(T)-F12b/VQZ-F12 geometries on formation of $H_3N\cdots XY$. Bond distances in Å and angles in degrees

XY	ΔR(X–Y)	ΔR(N–H)	$\Delta\theta$(HNH)
F_2	+0.013	0.000	+0.19
Cl_2	+0.026	0.000	+0.71
ClF	+0.059	−0.001	+1.95

3 Z-matrices from basis set convergence study

3.1 AVDZ

$H_3N\cdots F_2$

```
f
f  1 1.41935648
x  2 1.0        1 90.0
n  2 2.68376480 3 90.0         1 180.0
h  4 1.01417814 2 112.17701667 3 180.0
h  4 1.01417814 2 112.17701667 5 120.0
h  4 1.01417814 2 112.17701667 5 240.0
```

$H_3N\cdots Cl_2$

```
cl
cl 1 2.01513367
x  2 1.0        1 90.0
n  2 2.70042062 3 90.0         1 180.0
h  4 1.01407236 2 111.73836111 3 180.0
h  4 1.01407236 2 111.73836111 5 120.0
h  4 1.01407236 2 111.73836111 5 240.0
```

$H_3N\cdots ClF$

```
f
cl 1 1.68216274
x  2 1.0        1 90.0
n  2 2.31603100 3 90.0         1 180.0
h  4 1.01345146 2 110.56223710 3 180.0
h  4 1.01345146 2 110.56223710 5 120.0
h  4 1.01345146 2 110.56223710 5 240.0
```

3.2 AVTZ

$H_3N\cdots F_2$

```
f
f  1 1.42281131
x  2 1.0        1 90.0
n  2 2.65699475 3 90.0         1 180.0
h  4 1.01249235 2 112.07048043 3 180.0
h  4 1.01249235 2 112.07048043 5 120.0
h  4 1.01249235 2 112.07048043 5 240.0
```

$H_3N\cdots Cl_2$

```
cl
cl 1 2.01833443
x  2 1.0        1 90.0
n  2 2.67354229 3 90.0         1 180.0
h  4 1.01242228 2 111.60276678 3 180.0
h  4 1.01242228 2 111.60276678 5 120.0
h  4 1.01242228 2 111.60276678 5 240.0
```

$H_3N\cdots ClF$

```
f
cl 1 1.68665263
x  2 1.0        1 90.0
n  2 2.29816788 3 90.0         1 180.0
h  4 1.01182794 2 110.42949432 3 180.0
h  4 1.01182794 2 110.42949432 5 120.0
h  4 1.01182794 2 110.42949432 5 240.0
```

3.3 AVQZ

$H_3N\cdots F_2$

```
f
f  1 1.42171100
x  2 1.0        1 90.0
n  2 2.65012340 3 90.0         1 180.0
h  4 1.01207811 2 112.02383956 3 180.0
h  4 1.01207811 2 112.02383956 5 120.0
h  4 1.01207811 2 112.02383956 5 240.0
```

$H_3N\cdots Cl_2$

```
cl
cl 1 2.01655066
x  2 1.0        1 90.0
n  2 2.66444491 3 90.0         1 180.0
h  4 1.01205802 2 111.53894748 3 180.0
h  4 1.01205802 2 111.53894748 5 120.0
h  4 1.01205802 2 111.53894748 5 240.0
```

$H_3N\cdots ClF$

```
f
cl 1 1.68677486
x  2 1.0        1 90.0
n  2 2.28929977 3 90.0         1 180.0
h  4 1.01147728 2 110.35555507 3 180.0
h  4 1.01147728 2 110.35555507 5 120.0
h  4 1.01147728 2 110.35555507 5 240.0
```

3.4 VDZ-F12

$H_3N\cdots F_2$

```
f
f  1 1.42457485
x  2 1.0        1 90.0
n  2 2.67801359 3 90.0         1 180.0
h  4 1.01242828 2 112.09125631 3 180.0
h  4 1.01242828 2 112.09125631 5 120.0
h  4 1.01242828 2 112.09125631 5 240.0
```

$H_3N\cdots Cl_2$

```
cl
cl 1 2.01069849
x  2 1.0        1 90.0
n  2 2.70107196 3 90.0         1 180.0
h  4 1.01237646 2 111.68392912 3 180.0
h  4 1.01237646 2 111.68392912 5 120.0
h  4 1.01237646 2 111.68392912 5 240.0
```

$H_3N\cdots ClF$

```
f
cl 1 1.68357591
x  2 1.0        1 90.0
n  2 2.31017864 3 90.0         1 180.0
h  4 1.01177592 2 110.50720169 3 180.0
h  4 1.01177592 2 110.50720169 5 120.0
h  4 1.01177592 2 110.50720169 5 240.0
```

3.5 VTZ-F12

$H_3N\cdots F_2$

```
f
f  1 1.42331826
x  2 1.0        1 90.0
n  2 2.65775222 3 90.0         1 180.0
h  4 1.01236106 2 112.04793567 3 180.0
h  4 1.01236106 2 112.04793567 5 120.0
h  4 1.01236106 2 112.04793567 5 240.0
```

$H_3N\cdots Cl_2$

```
cl
cl 1 2.01688064
x  2 1.0        1 90.0
n  2 2.67566124 3 90.0         1 180.0
h  4 1.01232264 2 111.59113990 3 180.0
h  4 1.01232264 2 111.59113990 5 120.0
h  4 1.01232264 2 111.59113990 5 240.0
```

$H_3N\cdots ClF$

```
f
cl 1 1.68611458
x  2 1.0        1 90.0
n  2 2.29956521 3 90.0         1 180.0
h  4 1.01172827 2 110.41926320 3 180.0
h  4 1.01172827 2 110.41926320 5 120.0
h  4 1.01172827 2 110.41926320 5 240.0
```

3.6 VQZ-F12

$H_3N\cdots F_2$

```
f
f  1 1.42214410
x  2 1.0        1 90.0
n  2 2.64959519 3 90.0         1 180.0
h  4 1.01204877 2 112.00604956 3 180.0
h  4 1.01204877 2 112.00604956 5 120.0
h  4 1.01204877 2 112.00604956 5 240.0
```

$H_3N\cdots Cl_2$

```
cl
cl 1 2.01614831
x  2 1.0        1 90.0
n  2 2.66417150 3 90.0         1 180.0
h  4 1.01198905 2 111.52584752 3 180.0
h  4 1.01198905 2 111.52584752 5 120.0
h  4 1.01198905 2 111.52584752 5 240.0
```

$H_3N\cdots ClF$

```
f
cl 1 1.68665875
x  2 1.0        1 90.0
n  2 2.29014648 3 90.0         1 180.0
h  4 1.01144431 2 110.34616308 3 180.0
h  4 1.01144431 2 110.34616308 5 120.0
h  4 1.01144431 2 110.34616308 5 240.0
```

4 CP-CCSD(T)-F12b/VTZ-F12 geometries

$H_3N\cdots F_2$

```
f
f  1 1.42331826
x  2 1.0        1 90.0
n  2 2.65775222 3 90.0         1 180.0
h  4 1.01236106 2 112.04793567 3 180.0
h  4 1.01236106 2 112.04793567 5 120.0
h  4 1.01236106 2 112.04793567 5 240.0
```

$H_3N\cdots Cl_2$

```
cl
cl 1 2.01688064
x  2 1.0        1 90.0
n  2 2.67566124 3 90.0         1 180.0
h  4 1.01232264 2 111.59113990 3 180.0
h  4 1.01232264 2 111.59113990 5 120.0
h  4 1.01232264 2 111.59113990 5 240.0
```

$H_3N\cdots ClF$

```
f
cl 1 1.68611458
x  2 1.0        1 90.0
n  2 2.29956521 3 90.0         1 180.0
h  4 1.01172827 2 110.41926320 3 180.0
h  4 1.01172827 2 110.41926320 5 120.0
h  4 1.01172827 2 110.41926320 5 240.0
```

$H_3N\cdots Br_2$

```
br
br 1 2.33369468
x  2 1.0        1 90.0
n  2 2.60314903 3 90.0         1 180.0
h  4 1.01217464 2 111.05921039 3 180.0
h  4 1.01217464 2 111.05921039 5 120.0
h  4 1.01217464 2 111.05921039 5 240.0
```

$H_3N\cdots BrCl$

```
cl
br 1 2.19362018
x  2 1.0        1 90.0
n  2 2.52912312 3 90.0         1 180.0
h  4 1.01215279 2 110.92192667 3 180.0
h  4 1.01215279 2 110.92192667 5 120.0
h  4 1.01215279 2 110.92192667 5 240.0
```

$H_3N\cdots BrF$

```
f
br 1 1.82075002
x  2 1.0        1 90.0
n  2 2.33259885 3 90.0         1 180.0
h  4 1.01207439 2 110.43342082 3 180.0
h  4 1.01207439 2 110.43342082 5 120.0
h  4 1.01207439 2 110.43342082 5 240.0
```

$H_3N\cdots I_2$

```
i
i  1 2.72037625
x  2 1.0        1 90.0
n  2 2.76656589 3 90.0         1 180.0
h  4 1.01258568 2 111.25867407 3 180.0
h  4 1.01258568 2 111.25867407 5 120.0
h  4 1.01258568 2 111.25867407 5 240.0
```

$H_3N \cdots IBr$

```
br
i  1 2.53146664
x  2 1.0        1 90.0
n  2 2.65418328 3 90.0         1 180.0
h  4 1.01258378 2 111.01686032 3 180.0
h  4 1.01258378 2 111.01686032 5 120.0
h  4 1.01258378 2 111.01686032 5 240.0
```

$H_3N \cdots ICl$

```
cl
i  1 2.38269153
x  2 1.0        1 90.0
n  2 2.60471181 3 90.0         1 180.0
h  4 1.01262204 2 110.96949762 3 180.0
h  4 1.01262204 2 110.96949762 5 120.0
h  4 1.01262204 2 110.96949762 5 240.0
```

$H_3N \cdots IF$

```
f
i  1 1.95944976
x  2 1.0        1 90.0
n  2 2.49574288 3 90.0         1 180.0
h  4 1.01261036 2 110.82492252 3 180.0
h  4 1.01261036 2 110.82492252 5 120.0
h  4 1.01261036 2 110.82492252 5 240.0
```

$H_3N \cdots At_2$

```
at
at 1 2.90019048
x  2 1.0        1 90.0
n  2 2.78242126 3 90.0         1 180.0
h  4 1.01267189 2 111.29020560 3 180.0
h  4 1.01267189 2 111.29020560 5 120.0
h  4 1.01267189 2 111.29020560 5 240.0
```

$H_3N \cdots AtI$

```
i
at 1 2.81802794
x  2 1.0        1 90.0
n  2 2.74107985 3 90.0         1 180.0
h  4 1.01273764 2 111.25863791 3 180.0
h  4 1.01273764 2 111.25863791 5 120.0
h  4 1.01273764 2 111.25863791 5 240.0
```

$H_3N \cdots AtBr$

```
br
at 1 2.62507847
x  2 1.0        1 90.0
n  2 2.66603767 3 90.0         1 180.0
h  4 1.01275950 2 111.14340059 3 180.0
h  4 1.01275950 2 111.14340059 5 120.0
h  4 1.01275950 2 111.14340059 5 240.0
```

$H_3N\cdots AtCl$

```
cl
at 1 2.47852991
x  2 1.0        1 90.0
n  2 2.63020774 3 90.0         1 180.0
h  4 1.01279327 2 111.13675166 3 180.0
h  4 1.01279327 2 111.13675166 5 120.0
h  4 1.01279327 2 111.13675166 5 240.0
```

$H_3N\cdots AtF$

```
f
at 1 2.05739701
x  2 1.0        1 90.0
n  2 2.55915379 3 90.0         1 180.0
h  4 1.01274082 2 111.09509332 3 180.0
h  4 1.01274082 2 111.09509332 5 120.0
h  4 1.01274082 2 111.09509332 5 240.0
```

5 Interaction and stabilisation energies

Table S3: Counterpoise corrected CCSD(T)-F12b interaction energies (kcal mol^{-1}) of $H_3N\cdots XY$ halogen bonding complexes. All single point energy calculations were performed on CCSD(T)-F12b/VTZ-F12 optimised geometries. Experimental intermolecular force constants k_σ (N m^{-1}) are listed for reference

XY	VDZ-F12	VTZ-F12	VQZ-F12	CBS	Expt. k_σ
F_2	-1.68	-1.74	-1.79	-1.83	4.7[34]
Cl_2	-4.54	-4.75	-4.87	-4.95	12.7[33]
ClF	-10.95	-11.24	-11.53	-11.64	34.3[35]
Br_2	-7.28	-7.50	-7.68	-7.79	18.5[36]
BrCl	-9.01	-9.31	-9.55	-9.67	26.7[37]
BrF	-15.89	-16.22	-16.50	-16.65	—
I_2	-7.64	-7.86	-8.07	-8.20	—
IBr	-10.36	-10.63	-10.90	-11.04	—
ICl	-12.11	-12.45	-12.75	-12.91	30.4[38]
IF	-17.15	-17.49	-17.78	-17.94	—
At_2	-9.29	-9.48	-9.67	-9.80	—
AtI	-10.66	-10.87	-11.10	-11.24	—
AtBr	-13.44	-13.70	-13.96	-14.10	—
AtCl	-15.24	-15.55	-15.84	-15.99	—
AtF	-19.63	-19.92	-20.17	-20.32	—

Table S4: Counterpoise corrected CCSD(T)-F12b stabilisation energies (kcal mol^{-1}) of $H_3N\cdots XY$ halogen bonding complexes. All single point energy calculations were performed on CCSD(T)-F12b/VTZ-F12 optimised geometries

XY	VDZ-F12	VTZ-F12	VQZ-F12	CBS
F_2	-1.64	-1.69	-1.73	-1.76
Cl_2	-4.33	-4.59	-4.70	-4.77
ClF	-9.83	-10.16	-10.40	-10.52
Br_2	-6.90	-7.15	-7.31	-7.40
BrCl	-8.38	-8.75	-8.95	-9.06
BrF	-14.76	-15.13	-15.39	-15.53
I_2	-7.38	-7.62	-7.79	-7.90
IBr	-9.89	-10.19	-10.41	-10.54
ICl	-11.44	-11.84	-12.10	-12.24
IF	-16.42	-16.80	-17.06	-17.22
At_2	-9.08	-9.26	-9.43	-9.54
AtI	-10.36	-10.57	-10.77	-10.90
AtBr	-12.97	-13.25	-13.47	-13.60
AtCl	-14.60	-14.97	-15.22	-15.36
AtF	-18.98	-19.33	-19.56	-19.71

6 NBO derived charge transfer

Table S5: Fraction of an electronic charge transferred from H_3N to XY on formation of the $H_3N\cdots XY$ halogen bond. Total charges calculated using the NBO method based on the CCSD/aug-cc-pV(T+d)Z density matrix

XY	Charge transferred
F_2	0.012
Cl_2	0.045
ClF	0.140
Br_2	0.085
BrCl	0.101
BrF	0.159
I_2	0.079
IBr	0.104
ICl	0.114
IF	0.135
At_2	0.091
AtI	0.099
AtBr	0.119
AtCl	0.127
AtF	0.138

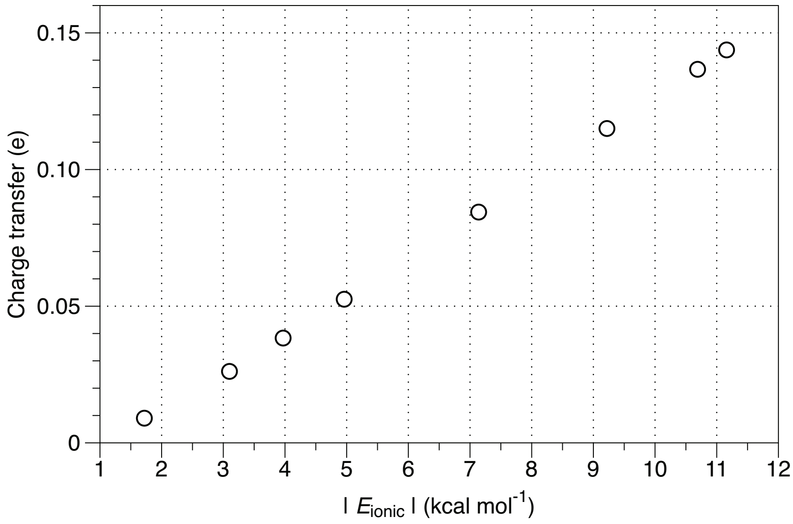

Figure S2: The fraction of an electronic charge transferred from H_3N to ClF as the angle θ is changed, plotted again the magnitude of the ionic substitution contribution to the LMP2/AVQZ interaction energy (E_{ionic}). Compare with Fig. 6 in the main manuscript.

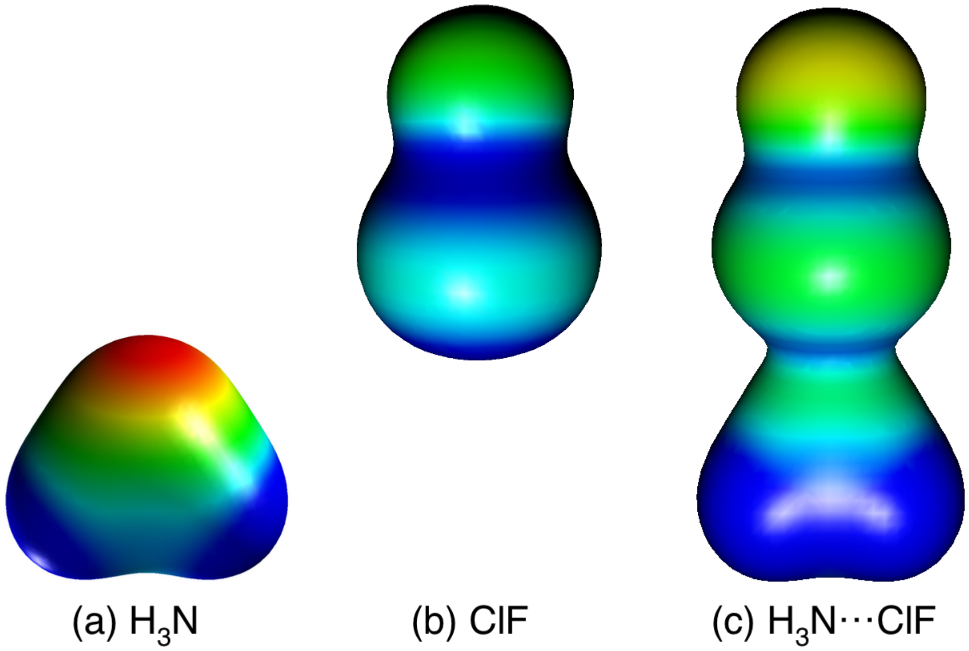

Figure S3: Isodensity surfaces (0.02 a.u.) colour-coded with the MP2/aug-cc-pVDZ electrostatic potential. Red indicates an electrostatic potential less than −0.1 a.u., blue greater than +0.1 a.u., and green between −0.05 and +0.05 a.u. Plots created using MOLDEN.[39]

7 $H_3N\cdots BrCl$ interaction energy partitioning

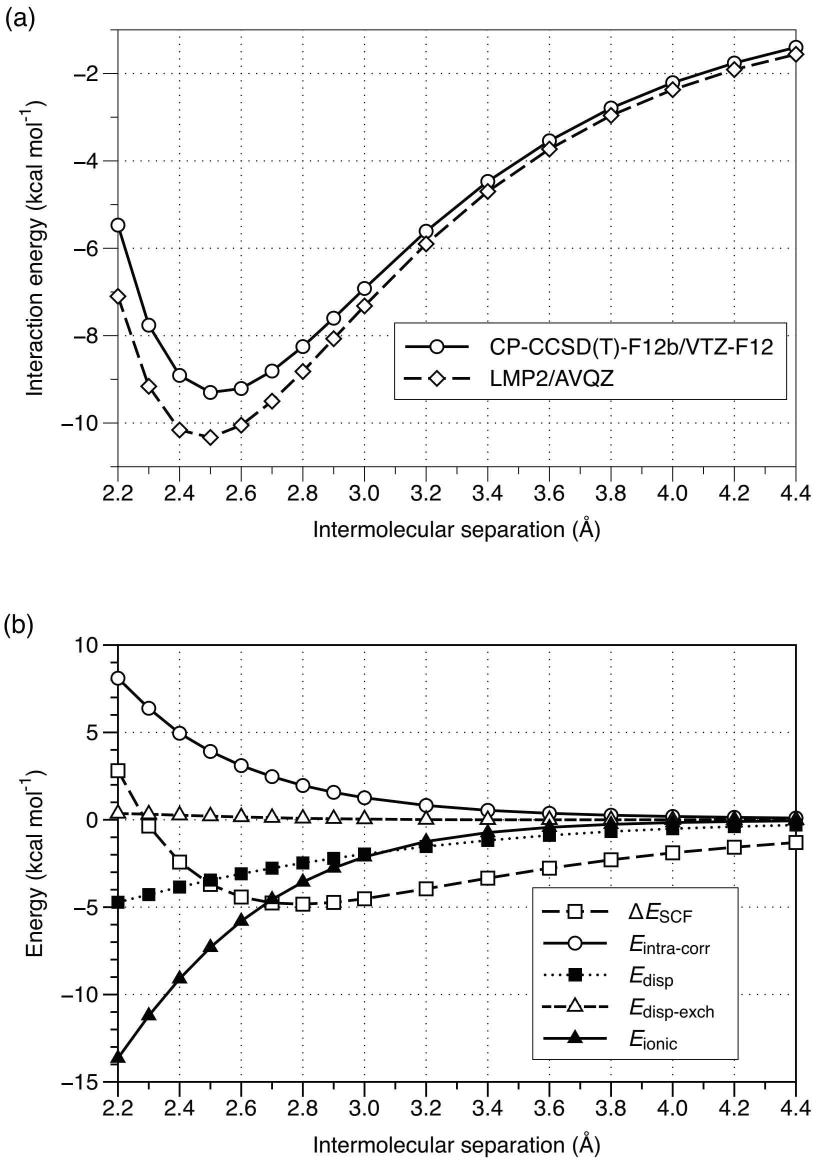

Figure S4: Potential energy scan of the intermolecular separation in the $H_3N\cdots BrCl$ halogen bonded complex. (a) comparison of the CP-CCSD(T)-F12b/VTZ-F12 and LMP2/AVQZ interaction energies. (b) partitioning of the LMP2/AVQZ interaction energy. All other bond lengths and angles are fixed at the CP-CCSD(T)-F12b/VTZ-F12 equilibrium geometry.

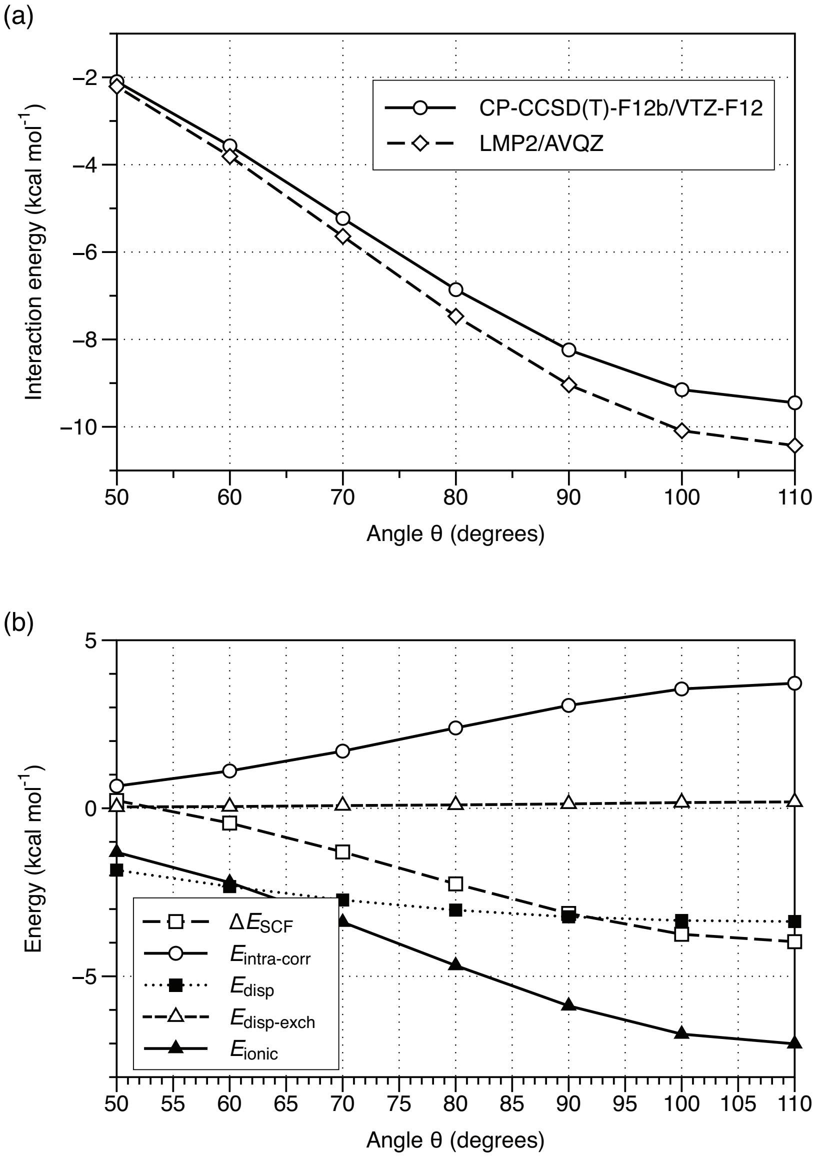

Figure S5: Potential energy scan of the H–N–Cl angle (θ) in the $H_3N\cdots BrCl$ halogen bonded complex. (a) comparison of the CP-CCSD(T)-F12b/VTZ-F12 and LMP2/AVQZ interaction energies. (b) partitioning of the LMP2/AVQZ interaction energy. All other geometrical parameters optimised, at each point, at the CP-CCSD(T)-F12b/VTZ-F12 level.

References

[1] H.-J. Werner, P. J. Knowles, G. Knizia, F. R. Manby, M. Schütz, et al., MOLPRO, version 2010.1, a package of ab initio programs, see http://www.molpro.net.

[2] H.-J. Werner, P. J. Knowles, G. Knizia, F. R. Manby, M. Schütz, *WIREs Comput. Mol. Sci.* **2012**, *2*, 242.

[3] T. B. Adler, G. Knizia, H.-J. Werner, *J. Chem. Phys.* **2007**, *127*, 221106.

[4] G. Knizia, T. B. Adler, H.-J. Werner, *J. Chem. Phys.* **2009**, *130*, 054104.

[5] S. Ten-no, *Chem. Phys. Lett.* **2004**, *398*, 56.

[6] K. A. Peterson, T. B. Adler, H.-J. Werner, *J. Chem. Phys.* **2008**, *128*, 084102.

[7] R. A. Kendall, T. H. Dunning Jr., R. J. Harrison, *J. Chem. Phys.* **1992**, *96*, 6796.

[8] T. H. Dunning Jr., K. A. Peterson, A. K. Wilson, *J. Chem. Phys.* **2001**, *114*, 9244.

[9] K. A. Peterson, J. G. Hill, **in preparation**.

[10] B. Metz, H. Stoll, M. Dolg, *J. Chem. Phys.* **2000**, *113*, 2563.

[11] K. A. Peterson, D. Figgen, E. Goll, H. Stoll, M. Dolg, *J. Chem. Phys.* **2003**, *119*, 11113.

[12] F. Weigend, *J. Comput. Chem.* **2008**, *29*, 167.

[13] F. Weigend, *Phys. Chem. Chem. Phys.* **2002**, *4*, 4285.

[14] F. Weigend, A. Köhn, C. Hättig, *J. Chem. Phys.* **2002**, *116*, 3175.

[15] C. Hättig, *Phys. Chem. Chem. Phys.* **2005**, *7*, 59.

[16] K. E Yousaf, K. A. Peterson, *J. Chem. Phys.* **2008**, *129*, 184108.

[17] K. E Yousaf, K. A. Peterson, *Chem. Phys. Lett.* **2009**, *476*, 303.

[18] E. F. Valeev, *Chem. Phys. Lett.* **2004**, *395*, 190.

[19] J. G. Hill, K. A. Peterson, G. Knizia, H.-J. Werner, *J. Chem. Phys.* **2009**, *131*, 194105.

[20] S. F. Boys, F. Bernardi, *Mol. Phys.* **1970**, *19*, 553.

[21] S. Simon, M. Duran, J. J. Dannenberg, *J. Chem. Phys.* **1996**, *105*, 11024.

[22] K. Szalewicz, B. Jeziorski, *J. Chem. Phys.* **1998**, *109*, 1198.

[23] T. Korona, D. Kats, M. Schütz, T. B. Adler, Y. Liu, H.-J. Werner in *Linear-Scaling Techniques in Computational Chemistry and Physics*, (Eds.: R. Zaleśny, M. G. Papadopoulos, P. G. Mezey, J. Leszczynski), Springer, Berlin, **2011**, p. 345.

[24] H.-J. Werner, F. R. Manby, P. J. Knowles, *J. Chem. Phys.* **2003**, *118*, 8149.

[25] R. Polly, H.-J. Werner, F. R. Manby, P. J. Knowles, *Mol. Phys.* **2004**, *102*, 2311.

[26] K. A. Peterson, *J. Chem. Phys.* **2003**, *119*, 11099.

[27] K. A. Peterson, B. C. Shepler, D. Figgen, H. Stoll, *J. Phys. Chem. A* **2006**, *110*, 13877.

[28] C. Hättig, G. Schmitz, J Koßmann, *Phys. Chem. Chem. Phys.* **2012**, *14*, 6549.

[29] J. Pipek, P. G. Mezey, *J. Chem. Phys.* **1989**, *90*, 4916.

[30] J. W. Boughton, P. Pulay, *J. Comput. Chem.* **1993**, *14*, 736.

[31] M. Schütz, G. Rauhut, H.-J. Werner, *J. Phys. Chem. A* **1998**, *102*, 5996.

[32] A. Karpfen in *Halogen bonding: fundamentals and applications*, (Eds.: P. Metrangolo, G. Resnati), Struct. Bond. Springer, Berlin, **2008**, p. 1.

[33] A. C. Legon, D. G. Lister, J. C. Thorn, *J. Chem. Soc. Faraday Trans.* **1994**, *90*, 3205.

[34] H. I. Bloemink, K. Hinds, J. H. Holloway, A. C. Legon, *Chem. Phys. Lett.* **1995**, *245*, 598.

[35] H. I. Bloemink, K. Hinds, J. H. Holloway, A. C. Legon, *Chem. Phys. Lett.* **1996**, *248*, 260.

[36] H. I. Bloemink, A. C. Legon, *J. Chem. Phys.* **1995**, *103*, 876.

[37] H. I. Bloemink, A. C. Legon, J. C. Thorn, *J. Chem. Soc. Faraday Trans.* **1995**, *91*, 781.

[38] E. R. Waclawik, A. C. Legon, *Phys. Chem. Chem. Phys.* **1999**, *1*, 4695.

[39] G. Schaftenaar, J. H. Noordik, *J. Comput.-Aided Mol. Design* **2000**, *14*, 123.
